# Carbuncle Treated by Cut Cups

**Published:** 1872-01

**Authors:** 


Carbuncle Treated by Cut Cups.—M. Hamon (*Bulletin Générale*, from the *Revue Méd.*, June, 1870,) recommends the application of cups to carbuncles after they have been freely lanced. From two to three ounces of blood should be removed in this way. He claims that the phlegmonous affection may in this way be aborted, if done in time, and that the sloughing and profuse suppuration, which are the consequences of the modes of treatment most in vogue, are avoided by it.

